# A global assessment of surveillance methods for dominant malaria vectors

**DOI:** 10.1038/s41598-021-94656-w

**Published:** 2021-07-28

**Authors:** Bram van de Straat, Tanya L. Russell, Kyran M. Staunton, Marianne E. Sinka, Thomas R. Burkot

**Affiliations:** 1 Australian Institute of Tropical Health and Medicine, James Cook University, Cairns, Australia; 2 Department of Zoology, University of Oxford, Oxford, UK

**Keywords:** Malaria, Entomology

## Abstract

The epidemiology of human malaria differs considerably between and within geographic regions due, in part, to variability in mosquito species behaviours. Recently, the WHO emphasised stratifying interventions using local surveillance data to reduce malaria. The usefulness of vector surveillance is entirely dependent on the biases inherent in the sampling methods deployed to monitor mosquito populations. To understand and interpret mosquito surveillance data, the frequency of use of malaria vector collection methods was analysed from a georeferenced vector dataset (> 10,000 data records), extracted from 875 manuscripts across Africa, the Americas and the Asia-Pacific region. Commonly deployed mosquito collection methods tend to target anticipated vector behaviours in a region to maximise sample size (and by default, ignoring other behaviours). Mosquito collection methods targeting both host-seeking and resting behaviours were seldomly deployed concurrently at the same site. A balanced sampling design using multiple methods would improve the understanding of the range of vector behaviours, leading to improved surveillance and more effective vector control.

## Introduction

Substantial progress has been made to reduce the global incidence of human malarias. As malaria transmission diminishes, malaria cases become more spatially heterogenous^1,2^. The latest World Health Organisation (WHO) guidance to national malaria programs encourages the use of local evidence to select interventions by transmission stratum, rather than utilising a one-size-fits-all approach^3^. Vector surveillance data is thus increasingly critical to support this informed decision-making process, with surveillance, including vector surveillance, now considered a core intervention^4^. The WHO recommends monitoring a set of vector surveillance indicators based on transmission intensity and vector control measures deployed^5–7^. These indicators are species identification, abundance, peak biting times, resting and biting locations, insecticide susceptibility and infection rates^6^. However, national programme capacity limitations (e.g. inadequate strategic frameworks, logistics shortfalls, limitations in human resources or financial constraints) often prohibit monitoring the complete set of indicators^8^. Guidance on which surveillance tools should be used to measure the recommended indicators are provided by the WHO. Each of these surveillance tools has associated biases and limitations^9^.

Globally, there are 41 dominant malaria vector species (DVS) responsible for most human malaria transmission. Each DVS has unique combinations of behaviours. In Africa, seven DVS are recognised^10^. Nine DVS are found in the Americas (North, Central and South America)^11^ and a staggering nineteen DVS are recognised in the Asia-Pacific region^12^. Additionally, species complexes containing cryptic species are found across all regions. One consequence of the regional variability in mosquito fauna is that the epidemiology of human malaria transmission and the effectiveness of vector control measures differs considerably between Africa, the Americas and the Asia-Pacific region^13,14^.

Representative vector surveillance requires unbiased sampling of mosquito populations. The high variability in biting and resting behaviours between regions and by vector species may impact the efficacy of sampling methods and potentially delay detecting changes in vector behaviours that reduce the efficacy of malaria control methods and thereby delay changing strategies to better control the vectors. Due to these differences in vector bionomics, certain patterns of behaviours are commonly associated with geographic areas. Many DVS in Africa are described as being distributed rurally, anthropophagic (preferring to bite humans) and predominantly biting late at night and indoors. Contrastingly, while also predominantly rural in distribution, many American and Asian DVS are considered to predominantly bite and rest outdoors and are opportunistic (more zoophagic) in their blood feeding preferences^10–12,15,16^. Exceptions abound: for example, *An. culicifacies* is an urban, indoor biting and resting mosquito found in Asia^17^ and *An. arabiensis* is an African vector that can exhibit opportunistic, outdoor blood feeding habits^18^. We hypothesise that these behavioural differences will impact collection method efficacy (i.e., the number of mosquitoes captured). Hence, collection methods might be selected on the basis of efficacy to maximise numbers captured. Thus, the use of traps might vary in the frequency with which they are deployed by geographic area. Consequently, the predominant vector behaviours reported in an area may also reflect the biases associated with the collection method used.

Here, the frequency of use of the most commonly deployed malaria vector collection methods in Africa, the Americas and the Asia-Pacific region between 1981 and 2015 was analysed from an extensive database of spatially defined information on anopheline vector bionomics^19^. Geographical patterns of collection method use from the three regions were compared with requirements for accurately monitoring malaria vectors.

## Results

**Figure 1:**
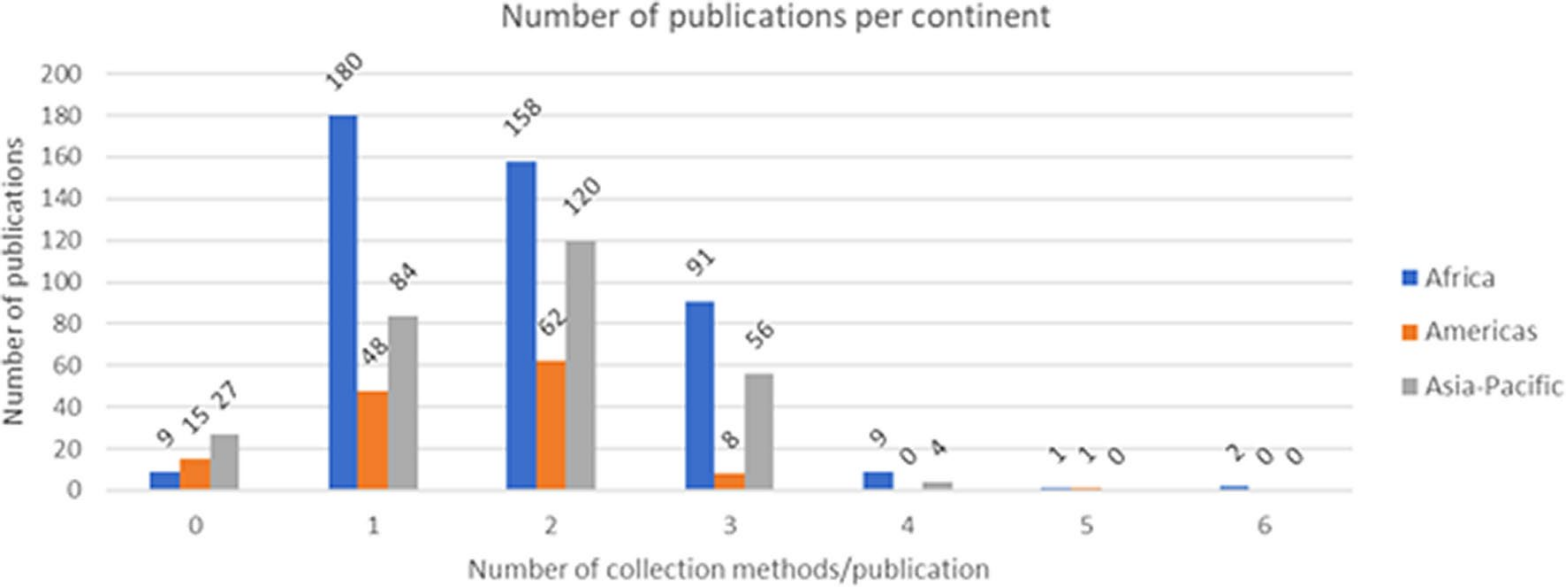
Number of publications per continent. For each continent, the number of publications is shown, as well as the number of collection methods that were reported in each publication. Zero collection methods used means that there was no specific information available about the type of collection method(s) used in a publication.

The database contained 5678 data records from 450 publications from Africa, 1346 data records from 134 publications from the Americas and 3898 data records from 291 publications from the Asia-Pacific region. Of these 875 publications, 51 (9 from Africa, 15 from the Americas, 27 from the Asia-Pacific region) did not contain specific information on collection methods. The majority of publications reported one (n = 312) or two (n = 340) collection methods used in the study, while three different collection methods were used in 155 publications (see Fig. 1). More than three collection methods were reported in only a few publications.

Individual data records (rows in the database) showed unique site-collection period-species combinations (see Supplementary Material 1 for an example). The count of collection methods used in Africa was 9824 with a mean number of 1.74 collection methods per data record. The count of collection methods used in the Americas was 1925 with a mean number of 1.47 collection methods per data record. The count of collection methods used in Asia-Pacific was 5819 with a mean number of 1.75 collection methods per data record. The different collection methods recorded in the database targeting host-seeking or resting mosquitoes were categorised as by Farlow et al.^9^, which showed that the same seven collection method groups were used in Africa, the Americas and the Asia-Pacific region (Tables 1, 2).

**Table 1:** Sampling methods for host-seeking mosquitoes.

Sampling method group	Definition/tool used	Sampling method (indoor)	Sampling method (outdoor)
Human landing catch	Mosquitoes attracted to collectors are captured by aspiration on exposed lower legs	HLC (Human landing catch) MB (Man biting, location unspecified) MBI (Man biting indoors)	MBO (Man biting outdoors)
Light trap	CO_2_-baited CDC trap with light bulb	ILT (Indoor light trap)	OLT (Outdoor light trap)
Human-baited double net trap	A collection method composed of two mesh tents with one inside the other. The inner tent contains and protects a human that acts as bait for host-seeking mosquitoes. The outer tent has a gap between the mesh wall bottom and the ground or open tent doors to allow mosquitoes to enter. Mosquitoes are trapped and retained between the tent walls and are periodically collected by aspiration		HBT (Human-baited tent) HBN (Human-baited net)
Animal-baited trap	As human-baited trap, but containing an animal (cow/goat/pig/monkey) instead of human	ABI (Animal-baited inside, in animal shelter) AB (Animal-baited, location unspecified)	CBT (Cow-baited trap) ABT (Animal-baited trap) ABO (Animal-baited outside) ABN (Animal-baited net trap)
Odour trap	A mechanical trap releasing CO_2_ and/or host odours while capturing attracted mosquitoes		Odour-bait

The different sampling methods recorded in Massey et al. (2016)^19^ targeting host-seeking mosquitoes were grouped into categories of techniques/tools used as proposed by Farlow et al. (2020)^9^.

**Table 2:** Sampling methods for resting and other behaviours.

Sampling method group	Definition/tool used	Sampling method (indoor)	Sampling method (outdoor)
Natural resting site collections	Capture of mosquitoes by oral, battery-powered and backpack aspirators; includes knockdown spray catches targeting resting mosquitoes in which an insecticide fog immobilises mosquitoes that fall to the floor and are collected	HRI (House resting indoors) RO (shelter)/RO (ani-shelter): resting in natural or animal shelters	RO (Resting outdoors)
Artificial resting site collections	Includes capture of mosquitoes in constructed shelters and Window Exit traps	WinExit: wire mesh (glue optional) covering the windows, which traps mosquitoes trying to exit houses	RO (pit): resting outdoors in pit traps and other man-made shelters like clay pots, resting boxes, barrier traps and Malaise traps

The different sampling methods recorded in Massey et al. (2016)^19^ targeting resting mosquitoes were grouped into categories of techniques/tools used as proposed by Farlow et al. (2020)^9^. Artificial resting sites are specifically constructed to lure and catch resting mosquitoes, while Natural resting sites are not, i.e. a house is not specifically constructed to lure and catch resting mosquitoes so it is considered a Natural resting site.

### Sampling host-seeking anophelines

Global analysis of the data showed that indoor and outdoor human landing catches (HLCs) were by far the most frequently used methods to collect anophelines. The proportional use of HLCs in the Asia-Pacific region was 0.597 (n_ASIA-PACIFIC_ = 3475) which was not significantly different to Africa at 0.604 (n_AFRICA_ = 5934) (Table 3). Contrastingly, the proportion of HLCs in the Americas was much higher, at 0.890 (n_AMERICAS_ = 1713). Comparing within continents, statistically there was no discernible preference for indoor or outdoor use of HLCs in Africa (HLC_OUT_ = 25.82%, HLC_IN_ = 31.03%; Mann–Whitney U, *p* = 0.28, n.s.), the Americas (HLC_OUT_ = 47.48%, HLC_IN_ = 33.82%; Mann–Whitney U, *p* = 0.85, n.s.) and the Asia-Pacific region (HLC_OUT_ = 29.20%, HLC_IN_ = 25.90%; Mann–Whitney U, *p* = 0.71, n.s.). However, when comparing between continents, outdoor HLCs tended to be more common in the Americas as well as the Asia-Pacific region when compared to Africa where there was a tendency to perform indoor HLCs, reflecting perceived vector biting characteristics (Table 3). Sampling location was not recorded for a small number of the HLCs (Africa: 3.55%, Americas: 7.69%, Asia-Pacific: 4.62%).

**Table 3:** The number of data records for each collection method used in Africa, the Americas and the Asia-Pacific region, categorised by 5-year period.

	HLC			Human-baited trap	Animal-baited trap	Light trap		Odour trap	Natural resting site collection		Resting (other)	Total
	Indoor	Outdoor	Unknown			Indoor	Outdoor		Indoor	Outdoor		
**Africa**												
1981–1985	71	22	1	0	0	2	1	0	12	0	2	111
1986–1990	512	351	61	19	16	124	6	0	332	35	84	1540
1991–1995	535	385	81	11	0	144	4	2	428	41	59	1690
1996–2000	545	541	58	15	0	85	1	9	762	17	24	2057
2001–2005	796	762	77	8	2	127	19	4	497	71	72	2435
2006–2010	537	448	38	4	5	156	69	1	344	7	95	1704
2011–2015	13	14	0	0	0	14	8	0	17	0	3	69
NA	0	0	0	0	1	0	0	0	0	0	0	1
Total	3048	2537	349	57	24	670	108	20	2489	173	349	9824
Prop.	31.03%	25.82%	3.55%	0.58%	0.24%	6.82%	1.10%	0.20%	25.34%	1.76%	3.55%	100%
**Americas**												
1981–1985	20	20	0	0	9	0	0	0	2	0	4	55
1986–1990	143	146	3	0	8	2	1	0	19	27	0	349
1991–1995	134	140	16	0	18	1	53	0	1	10	0	373
1996–2000	44	160	23	0	0	11	0	0	2	3	0	243
2001–2005	99	185	99	8	0	0	1	0	1	12	0	405
2006–2010	209	222	7	0	0	6	0	8	0	3	0	455
2011–2015	2	2	0	1	0	0	0	0	0	0	1	6
NA	0	39	0	0	0	0	0	0	0	0	0	39
Total	651	914	148	9	35	20	55	8	25	55	5	1925
Prop.	33.82%	47.48%	7.69%	0.46%	1.82%	1.04%	2.86%	0.42%	1.29%	2.86%	0.26%	100%
**Asia-Pacific region**												
1981–1985	204	233	0	4	91	0	8	0	98	22	0	660
1986–1990	450	366	152	23	10	43	44	0	308	72	5	1473
1991–1995	187	286	28	18	41	16	51	7	535	2	4	1175
1996–2000	418	494	8	0	27	31	19	1	195	47	1	1241
2001–2005	114	162	10	8	8	14	11	0	186	54	8	575
2006–2010	131	147	57	29	3	51	8	0	169	11	10	616
2011–2015	0	2	0	0	0	3	1	0	2	3	0	11
NA	3	9	14	15	6	4	0	0	14	0	3	68
Total	1507	1699	269	97	186	162	142	8	1507	211	31	5819
Prop.	25.90%	29.20%	4.62%	1.67%	3.20%	2.78%	2.44%	0.14%	25.90%	3.63%	0.53%	100%

The data frame was manipulated to summarise the combination of collection methods used per data record, ensuring that the location (indoors or outdoors) for each sampling effort was noted. When individual data records were examined in further detail, HLC collections were used in three sampling strategies: indoor-only, outdoor only and simultaneously indoor and outdoor. Simultaneous indoor and outdoor HLCs were common practice in all regions. In the analysed data, 77.4% of HLCs in Africa, 50.1% of HLCs in the Americas and 67.5% of HLCs in the Asia-Pacific region were deployed indoors and outdoors simultaneously. Interestingly, the proportion of outdoor-only HLC collections is much larger in the Americas and the Asia-Pacific region than in Africa (Americas: 21.1%, Asia-Pacific: 21.1%, Africa: 3.5%). The opposite is true for indoor-only HLC collections, which occurred more often in Africa (Africa: 19.1%, Americas: 0.6%, Asia-Pacific: 11.4%).

Indoor and outdoor light trap collections in Africa showed large differences (Table 3). Indoor light trap collections were deployed 6-times more frequently than outdoor light trap collections (light trap_IN_ = 6.82%, light trap_OUT_ = 1.10%, Mann–Whitney U, *p* = 0.03). Indoor and outdoor light trap collections in the Asia-Pacific region accounted for only 2.78% and 2.44% of total sampling effort, respectively, and did not significantly differ (Mann–Whitney U, *p* = 0.42, n.s.) (Table 3). In the Americas, indoor and outdoor light trap collections were deployed infrequently as well (light trap_IN_ = 1.04%, light trap_OUT_ = 2.86%, Mann–Whitney U, *p* = 0.28, n.s.). Indoor light trap collections were more often deployed in Africa than in the Asia-Pacific region, although the difference was not statistically significant (n_AFRICA_ = 670, n_ASIA_ = 162; Mann–Whitney U, *p* = 0.086).

Alternative sampling methods to HLCs designed to collect host-seeking mosquitoes—human-baited tent traps (HBTs) and animal-baited tent traps (ABTs)—were more commonly used in the Asia-Pacific region than in Africa and the Americas, albeit infrequently (Table 3). Animal-baited trap use accounted for 3.2% (n = 186), while HBT use accounted for only 2.0% (n = 97) of the total sampling effort in the Asia-Pacific region. The use of ABTs in the Asia-Pacific region decreased strongly after the year 2000, while relative HBT use varied strongly between the 5-year periods. In Africa, HBTs were rarely used (n = 57). Their use peaked at 0.6% in 1986–1990 and decreased thereafter with no data records of HBT use in the period 2011–2015. ABT deployment in Africa was even less common, accounting for only 0.24% of data records (n = 24), of which 16 occurred between 1986 and 1990. ABT and HBT use was also uncommon in the Americas (n_ABT_ = 35, n_HBT_ = 9), where the deployment of ABTs was not recorded in the database after 1995.

### Sampling resting anophelines

The collection of anophelines from natural indoor resting sites (defined as structures, including houses and animal shelters, not constructed specifically to lure resting mosquitoes^20^) was the second most commonly used method in both the Asia-Pacific region (29.5%, n = 1718) and Africa (27.1%, n = 2662; Table 3). While the collection of anophelines from natural resting sites was also the second most frequently used method in the Americas, it was infrequently used (4.15%, n = 80). The ratio between indoor and outdoor collections was highly skewed in Africa and the Asia-Pacific region, with indoor resting collections exceeding outdoor resting collections from vegetation sevenfold in the Asia-Pacific region (nat. resting_OUT_ = 3.63%, nat. resting_IN_ = 25.90%; Mann–Whitney U, *p* = 0.01) to almost 15-fold in Africa (nat. resting_OUT_ = 1.76%, nat. resting_IN_ = 25.34%; Mann–Whitney U, *p* = 0.03). In the Americas, outdoor resting collections amongst vegetation exceeded indoor resting collections twofold but this was not significant (nat. resting_OUT_ = 2.86%, nat. resting_IN_ = 1.29%; Mann–Whitney U, *p* = 0.82). It is important to note that the small proportion of outdoor resting collections generally reflected the difficulty of collecting mosquitoes outdoors and not the absence of outdoor resting mosquitoes.

When sampling location per data record was taken into account, natural resting collections showed a sharp contrast in both Africa and the Asia-Pacific region, with indoor-only resting collections (Africa: n = 2248, 92.9%; Asia-Pacific region: n = 1325, 86.3%) far outnumbering simultaneous indoor and outdoor resting collections (Africa: n = 144, 5.9%; Asia-Pacific region: n = 168, 10.9%) as well as outdoor-only resting collections (Africa: n = 27, 1.1%; Asia-Pacific region: n = 43, 2.8%). The Americas contrasted with the other two geographic regions analysed, because outdoor-only resting collections from vegetation (n = 43, 63.2%) outnumbered indoor-only resting collections (n = 13, 19.1%) and simultaneous indoor and outdoor resting collections (n = 12, 17.6%).

Pit traps and other artificial resting sites (e.g. clay pots, box traps etc.) can be used as alternatives to vegetation aspiration to increase mosquito numbers in outdoor resting collections^21^. Only information on the use of pit traps was found in the dataset for artificial resting site collections. Pit traps were rarely used in the Americas (n = 4) and the Asia-Pacific region (n = 15), where aspiration of natural vegetation was more common. In Africa however, pit trap use was more frequent (n = 156) and comparable to the aspiration of vegetation (n = 173). Pit traps were used in 27 African studies included in the database and, in most of these studies, used in lieu of the aspiration of vegetation.

### Temporal patterns in sampling methods

**Figure 2:**
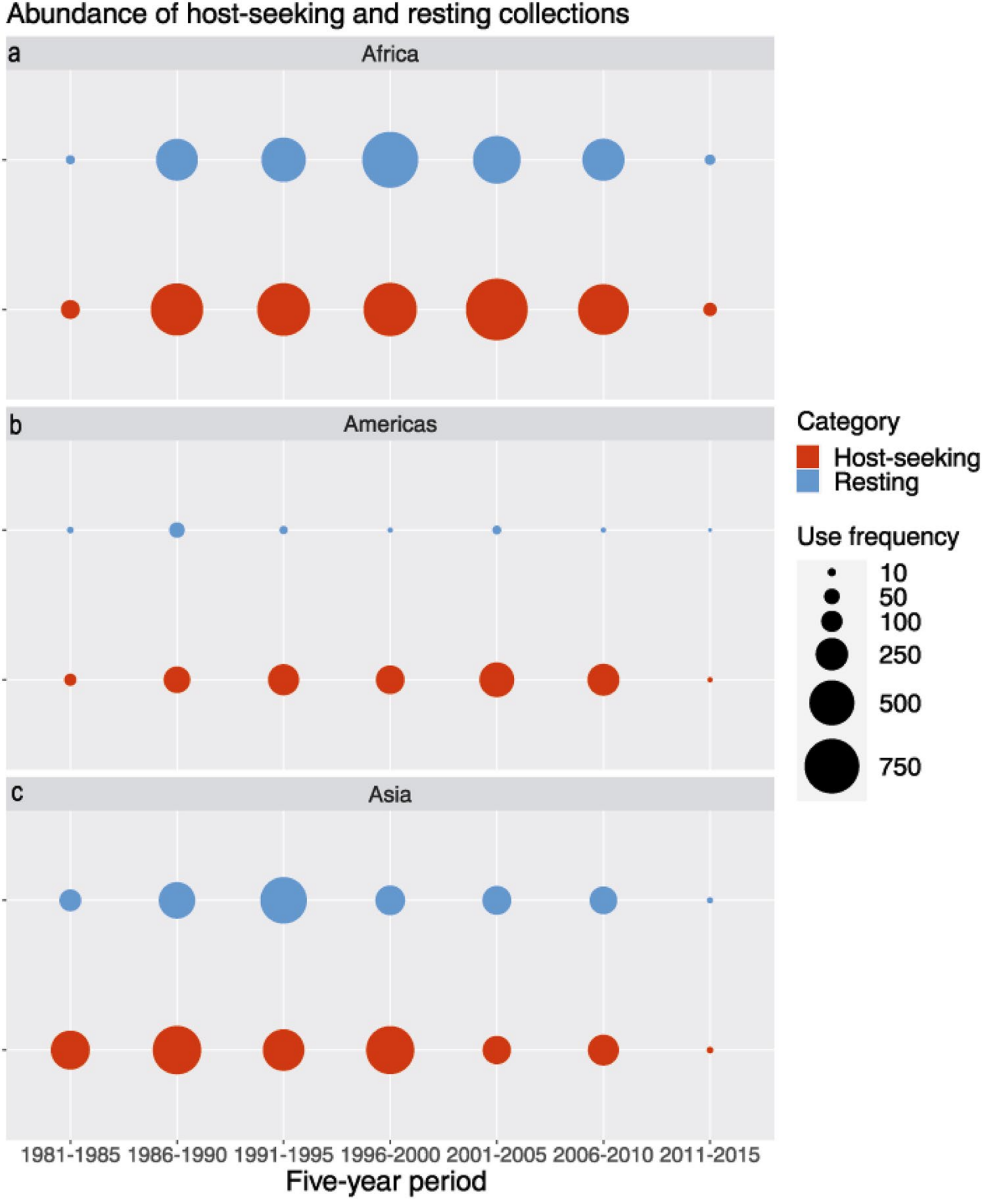
Number of data records. The number of data records for the two categories of collection methods, host-seeking and resting, presented per 5-year time period. (**a**) Africa, (**b**) Americas, (**c**) Asia-Pacific region.

In Africa, malaria vector sampling increased in the late 1980s for both host-seeking as well as resting collections (Fig. 2a). Total sampling effort remained quite stable in the following years to 2010. In the Americas (Fig. 2b), the abundance of host-seeking collections followed a similar pattern to Africa: between 1986 and 2010 the abundance of host-seeking collections remained quite stable. Resting collection sampling frequency, on the other hand, decreased in the 1990s and remained a very infrequently deployed sampling method until 2011–2015 (the final recorded period of the database). In the Asia-Pacific region, the abundance of host-seeking and resting collections varied more than in the other continents (Fig. 2c). Remarkably, the total sampling effort in the Asia-Pacific region was much lower in the twenty-first century than it was in the twentieth century, while the total sampling effort in Africa and the Americas did not show such a decrease. Between 2011 and 2015, sampling effort declined sharply in the three analysed regions, which is most likely due to the small number of published records.

**Figure 3:**
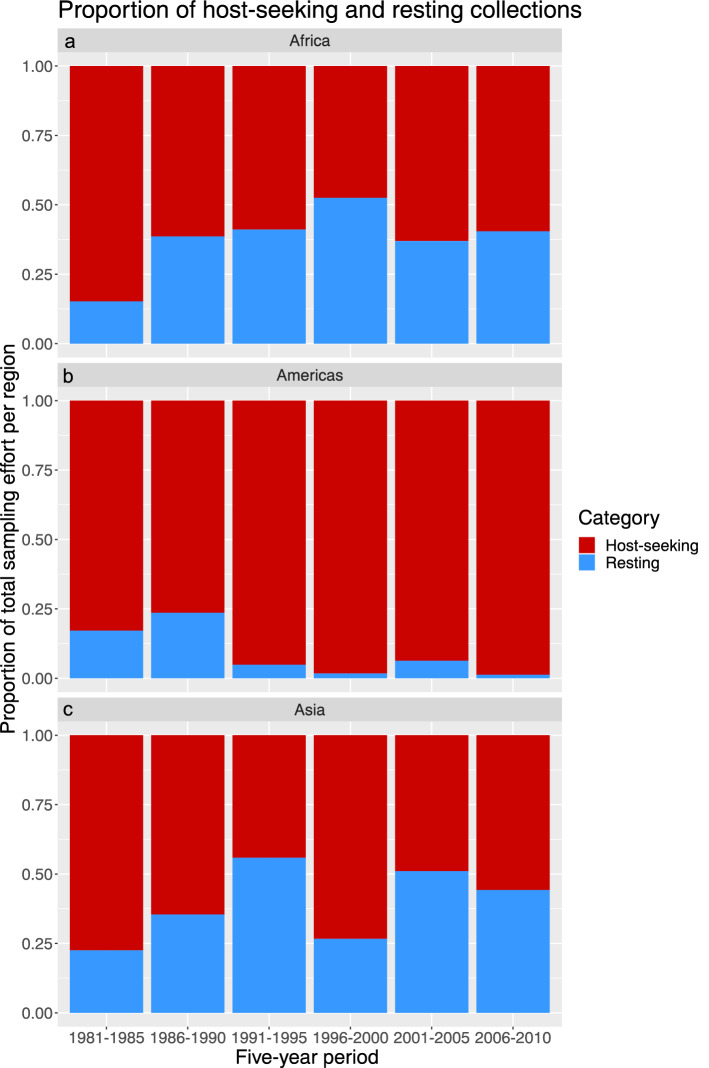
Proportion of host-seeking and resting collections. The proportion of host-seeking (red) and resting (blue) collections presented per 5 year time period for each region analysed. (**a**) Africa, (**b**) Americas, (**c**) Asia-Pacific region.

Although a slight upward trend can be seen in the proportion of resting collections, relative to host-seeking collections in Africa, this was by no means significant (Fig. 3a). The ratio between host-seeking and resting collections in Africa was the most stable of the three geographic areas analysed. The Americas was the only region in this analysis which showed a highly skewed ratio between host-seeking and resting collections (Fig. 3b). Here, resting collections were being used regularly in the 1980s, but the sampling effort in the Americas consisted almost entirely of host-seeking collections since the 1990s. In the Asia-Pacific region, the proportions of host-seeking and resting collections showed a slight upward trend but was not as stable as in Africa (Fig. 3c). The more varying ratios in the Asia-Pacific were not significantly different from the ratios observed in Africa (GLM, Tukey’s Post-hoc comparison, *p* > 0.99). However, the clear decrease in the proportion of resting collections since the 1990s in the Americas was significantly different from Africa (GLM, Tukey’s Post-hoc comparison, *p* = 0.0014) and the Asia-Pacific region (GLM, Tukey’s Post-hoc comparison, *p* = 0.0022). The variation in resting collections among the analysed periods was not significant (GLM, Tukey’s Post-hoc comparison, *p* > 0.65).

**Figure 4:**
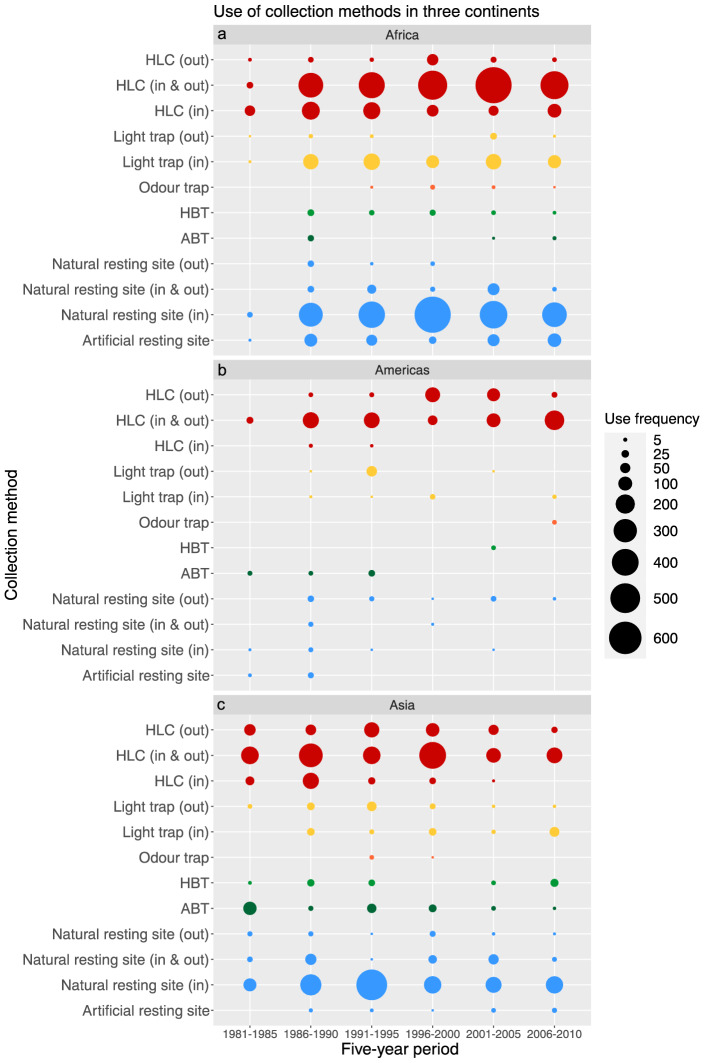
Number of data records per collection method. The number of data records for each collection method used in (**a**) Africa, (**b**) the Americas, (**c**) the Asia-Pacific region, presented per 5-year time period. ABT = animal-baited trap, HLC = human landing catch, HBT = human-baited double net trap.

The total sampling effort for each region is displayed in Fig. 4. In Africa, HLC (simultaneously indoors and outdoors) and indoor resting collections dominated the entire database time frame. Light traps were consistently deployed indoors, but were almost never deployed outdoors. Other alternative methods to HLCs, specifically HBTs, ABTs and odour traps, were used infrequently in Africa. In the Americas, HLCs made up the bulk of host-seeking mosquito collections. Sampling of natural resting sites occurred mainly outdoors since the 1990s, which contrasts with the indoor natural resting site collections in Africa and the Asia-Pacific region. Alternatives to HLC were rarely deployed. Together with the infrequent use of resting collections, this creates a highly singular dependence on surveillance data from HLCs. The Asia-Pacific region, like Africa, showed that surveillance data depended largely on HLCs and indoor resting collections. However, the deployment location of light traps was more evenly divided between indoors and outdoors than in Africa. Additionally, HBTs and ABTs were more often used as an alternative or complementary method to HLCs in the Asia-Pacific region than either in Africa or the Americas. Animal-baited traps were consistently used since 1980, whereas HBTs were used less frequently, and not at all in 1996–2000. What is also remarkable is that the overall mosquito sampling effort decreased in the periods 2001–2005 and 2006–2010 in the Asia-Pacific region, while sampling effort in Africa and the Americas remained stable.

### Geospatial patterns in sampling method use

**Figure 5:**
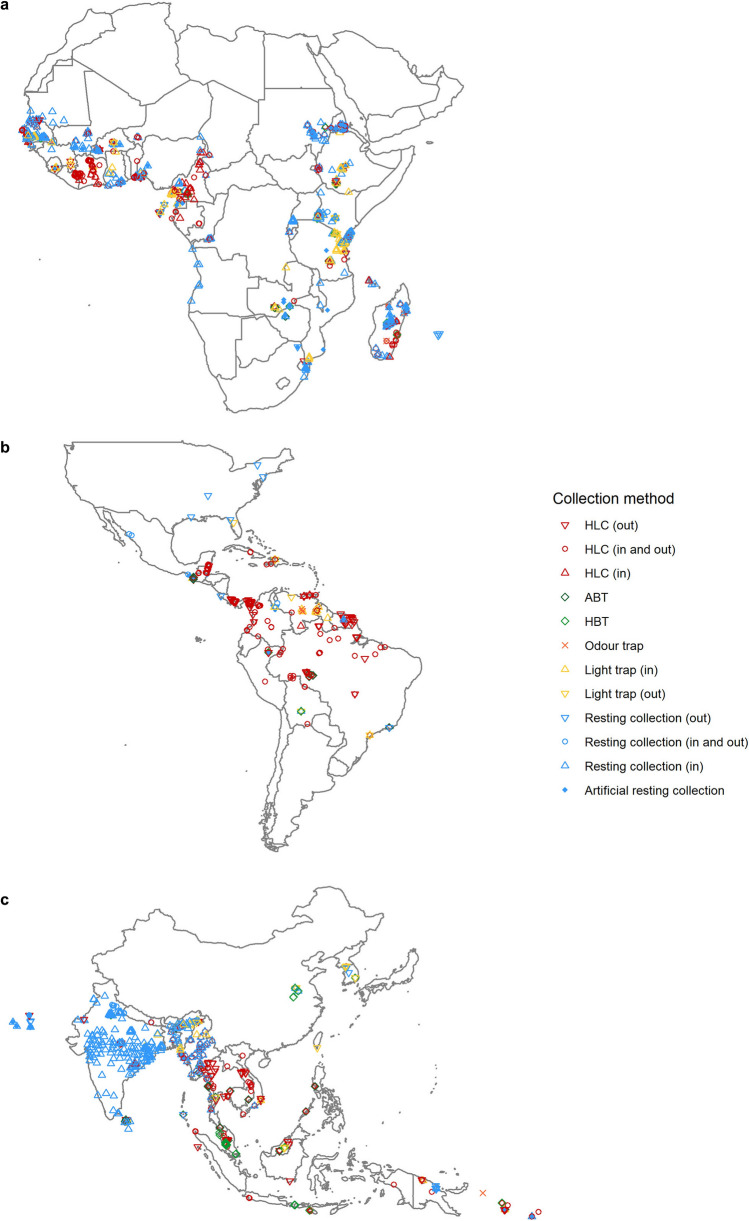
Geographical distributions. Geographical distribution of the different collection methods used in (**a**) Africa, (**b**) the Americas and (**c**) the Asia-Pacific region to collect malaria vectors. ABT = animal-baited trap, HLC = human landing catch, HBT = human-baited double net trap. Maps were made with R statistical software (R version 4.0.2), packages ‘tidyverse’ and ‘maps’^45^.

Visual inspection of the plotted locations of collection methods in the three analysed regions suggested that there was a clustering of collection method use in specific areas (Fig. 5). In Africa, the locations of indoor/outdoor HLCs and indoor natural resting collections were confined to West and East Africa, with very limited sampling in Central Africa (Fig. 5a). However, the data did not immediately show a geospatial pattern in the spread of both methods. 30 and 48 density-based clusters were detected of HLC (indoor/outdoor) and indoor natural resting collections, respectively. The detected clusters for both methods were spread across the continent, not being confined to one country and were mostly of small size, indicating that the sampling effort in these few areas was high. It is possible that these clusters roughly reflect the locations of research facilities. Computing Moran’s I for all the locations in Africa where surveys occurred supported the absence of continent-wide clustering of any sampling method (Moran’s I = 0.09, *p* > 0.95, n.s.).

In the Americas, the locations of indoor/outdoor HLCs were widespread across the region, with only a few locations of natural resting collections (Fig. 5b). The density-based cluster calculation detected six clusters of HLC (indoor/outdoor) locations, but these were not country-specific and additionally, five of the six clusters overlapped each other. Density-based cluster analyses were unable to detect any significant spatial clustering in other sampling methods due to the limited data. However, computing Moran’s I for all locations where sampling occurred indicated that there was spatial autocorrelation of sampling methods (Moran’s I = 0.19, *p* < 0.001).

In the Asia-Pacific region, a distinction could be seen in the spread of the sampling methods most often used (Fig. 5c). Indoor/outdoor HLCs were deployed across the entire Asia-Pacific region with the exception of India, where indoor natural resting collections were mainly deployed while sparsely used in the rest of the region. This was also shown by the density-based cluster detection, which detected 13 clusters of HLC (indoor/outdoor), of which the largest were not country-specific and were all found in the Greater Mekong Subregion (Myanmar, Thailand, Vietnam, Lao PDR and Cambodia). Smaller, country-specific clusters of indoor/outdoor HLCs were detected in the Philippines, Papua New Guinea and the Solomon Islands. Sixteen clusters of indoor natural resting collections were detected by density-based clustering, fourteen of which were (partially) in India. One large cluster was found in Myanmar and the final cluster was in Sri-Lanka. Outside these countries, indoor natural resting collections were too few in number and too wide-spread to fall into a cluster. Clustering of methods was supported by Moran’s I (Moran’s I = 0.27, *p* < 0.001), which showed that similar methods were more often deployed near each other than randomly spread.

## Discussion

This investigation analysed data from 875 distinct studies to examine collection methods use in Africa, the Americas and the Asia-Pacific region. Differences among the methods used to collect anophelines were observed among the regions and the potential causes for these differences explored. All analyses performed in this research were based on a precompiled bionomics dataset, which collated data from published studies. While the dataset was very rich, it did not contain data from national malaria control programmes (NMCPs) and other routine surveillance programs. Such documents are rarely publicly available and are often not published in English, which made finding these publications hard and data extraction unreliable. Therefore, we assumed that the dataset used for our analyses was a representative sample of the existing data on malaria vectors in Africa, the Americas and the Asia-Pacific region.

The HLC was the most commonly used method to sample mosquitoes in the three regions analysed. The HLC is considered the ‘gold standard’ in mosquito collections, because it gives the most accurate estimate of the human exposure to mosquito bites^9^. However, biting rates from HLCs are crude and likely overestimate true human biting rates since collectors, in contrast to the general public, do not make any attempts to ward off mosquitoes^21,22^. While concerns are often raised that collectors may be bitten by a mosquito before they can collect it, human landing collectors actually have a reduced risk of acquiring malaria due to prophylaxis^23^. Despite the reduced malaria risk, collectors are still vulnerable to arbovirus exposure and thus many attempts have been made to find an alternative to HLCs^24–30^. Despite its drawbacks, HLC still remains the most frequently used method for collecting malaria vectors.

The use of CDC light traps did not differ significantly between Africa, the Americas and the Asia-Pacific region. Outdoor light traps were not commonly deployed in any region, but indoor light traps were more often deployed in Africa than in the Americas or the Asia-Pacific region. Although this difference is not statistically significant, sampling more frequently indoors in Africa is consistent with the current dogma that most of the African DVS are endophagic^10,15^. The fact that the African DVS are perceived as endophagic and anthropophilic is consistent with the very limited use of animal-baited traps (24 data records from 6 studies) in this region. Additionally, bionomics data on African anophelines shows that their indoor/outdoor biting ratio is around 50/50^19^. By focussing on sampling indoors on human hosts, researchers might miss potentially important shifts in mosquito behaviours toward increased rates of opportunistic feeding or an increase in outdoor biting^31^. This might overestimate the perceived vulnerability of vectors to indoor interventions and thus overestimate the effectiveness of the currently recommended control methods, long-lasting insecticidal nets (LLINs) and indoor residual spraying of insecticides (IRS), that protect people from mosquito bites when they are indoors^32–34^.

Animal-baited traps were more commonly used in Asia-Pacific than in Africa or the Americas. However, this collection method was not as popular as expected when considering the perceived more opportunistic biting behaviour of many Asian vectors^12^, being only used in 25 out of 292 studies. The limited use of ABTs may reflect their significant operational constraints of being labour intensive and requiring substantial training for efficient deployment^7,9^. Hence, animal-baited traps were mainly used as a complementary method to HLC, instead of an alternative, thus covering a wider range of mosquito biting behaviours. Increasing the amount of entomological data from a wider array of surveillance methods for different vector behaviours is recommended for both researchers and NMCPs^6^. Animal-baited collection methods can play an important role in understanding vector behaviours more completely.

Natural resting site collection of anophelines was the second-most commonly used mosquito sampling method. This method encompasses indoor and outdoor handheld aspirators, backpack aspirator collections and pyrethrum spray collections (PSC). While indoor collections of resting mosquitoes are easier to conduct and yield reasonable sample numbers, outdoor collections of resting mosquitoes are notoriously difficult due to mosquitoes’ wide and heterogeneously dispersed resting sites^35,36^. This is consistent with observations in this study that showed that a large majority of resting collections (> 85% in Africa and the Asia-Pacific region) were conducted indoors. Although some inference on outdoor resting can be made by examining abdominal status of mosquitoes collected indoors^37–39^, the tendency to preferably collect resting mosquitoes by indoor sampling may have generated a bias towards indoor resting in our understanding of resting behaviours. Additionally, the tendency towards indoor resting collections may have delayed detection of changes in resting behaviours (e.g. behavioural resistance) following scale-up of indoor residual spraying. The current assumption, especially in Asia, is that the majority of malaria vectors rest outdoors^12^. Hence, surveillance strategies that only sample resting mosquitoes indoors will maintain the present knowledge gap on (outdoor) resting behaviours of malaria vectors.

Artificial resting sites (pit traps) were used in Africa as an alternative collection method to sample outdoor-resting anophelines. Other ways to create artificial resting sites for outdoor mosquito collections (clay pots, box shelters, etc.^21^) were not documented in the dataset analysed, so direct comparisons between respective sampling efforts of pit traps and other methods could not be made. However, Odiere et al. (2007)^35^ reported that clay pots were more successful and practical than pit traps in collecting both the more endophilic *An. gambiae* s.s. and the more exophilic *An. arabiensis*. In contrast, more recent research showed that clay pots were somewhat less productive than pit traps in collecting *An. gambiae* s.l.^40,41^. However, clay pots yielded a comparable relative abundance of anopheline species and were more practical in many situations, thereby offsetting their lower productivity. In areas with traditionally high or increased (e.g., resulting from high IRS coverage) levels of exophily, outdoor resting surveillance is crucial in understanding the ecology of malaria vectors and the effectivity of applied interventions. In such areas, not only in Africa but also in the Americas and the Asia-Pacific region, artificial resting site collections could be a valuable asset in vector surveillance.

The geospatial pattern of published mosquito collections in the Asia-Pacific region shows that HLCs, both indoor and outdoor, are the main surveillance method in the entire region except India where indoor resting collections are common practice. The astonishing number of data records from India (almost 50% of the data from the Asia-Pacific region comes from India) were almost exclusively acquired by indoor resting collections and may reflect an emphasis on one DVS, *Anopheles culicifacies*, which rests and bites traditionally indoors. However, expert opinions and bionomics data show that *An. culicifacies* can also be found resting outdoors^12,19^.

The use of a single method or insufficient sampling sites used to define a vector’s resting behaviours may limit the capacity to detect potential changes in its resting behaviour in response to selective pressure from indoor residual spraying or LLINs (e.g. resting indoors or outdoors, duration of resting at one location, peak biting time, endophagy/exophagy or choice of blood host)^12,42,43^. Most resting collections (mechanical aspirator collections, PSCs) are conducted in the early morning^21^ and cannot detect shifts in temporal or spatial resting patterns. Furthermore, data on host-seeking or biting behaviours cannot be derived from resting collections. A comparable argument can be made when HLCs alone are used to define biting behaviours. HLCs can detect spatio-temporal shifts in host-seeking and biting behaviours when contemporaneous indoor and outdoor collections are made, but cannot be analysed for changes in blood host choice^43^.

The use of one sampling method, specifically the method which results in the highest vector numbers, is a cost-effective way of acquiring large numbers of mosquitoes to define a limited number of vector indicators. In contrast, collecting mosquitoes by using multiple sampling methods for biting and resting, indoors and outdoors (and by representative sampling), yields more epidemiologically relevant data for researchers and policy makers. Therefore, entomological surveillance should utilise multiple complementary collection methods across different micro-habitats to sample different behaviours. Concurrent use of complementary collection methods will enable a more comprehensive characterisation of vector behaviours, will better define vector species richness and community composition, as well as enable the early detection of behavioural shifts that may threaten the effectiveness of malaria vector control^34^.

## Conclusions

We observed a tendency towards using collection methods to potentially maximise the number of mosquitoes captured based on anticipated vector behaviours in a geographic region by targeting specific vector behaviours (and by default, ignoring other behaviours). Although similar malaria vector collection methods were used in the three regions, their frequency of use varied between Africa, the Americas and the Asia-Pacific region. Their frequency of use may have resulted from biases in the perceived behaviours of the DVS in each region in order to maximise the numbers captured. Adherence to current dogmas and expert opinions to design vector sampling strategies may reinforce biases in surveillance data and can delay the detection of behavioural shifts of vectors which could lead to reduced vector control. A more varied, tailor-made surveillance effort integrating multiple collection methods for specific regions can provide better insights in vector behaviour and changes in vector behaviours.

## Methods

All analyses in this study were based on the bionomics data extracted and collated from literature published between 1981 and 2015 on the global DVS^19^. A detailed structure of the database as well as original references are in Supplementary File 1 and the original publication. The database contained 10,922 data records from 875 publications. Information on *Anopheles* species included the (georeferenced) study location, starting month and year of the study, mosquito collection methods used, biting and resting locations, preferred blood hosts and peak biting times. The methods used to collect malaria vectors in the WHO regions ‘Africa’, ‘Americas’ and ‘Asia-Pacific’ were analysed because 96.4% of the global malaria burden is found in these regions^5^, where malaria vector behaviours differ.

Individual data records (rows in the database) show unique site-collection period-species combinations. A single study or publication could therefore comprise multiple data records (rows), depending on: (1) the number of sites studied, (2) the number of sampling events included in the study, (3) the commencement date and (4) the species collected. Collection sites were recorded with geographical latitude and longitude, without differentiating between the area size, which included “point” (≤ 10 km^2^), “wide-area” (> 10 and ≤ 25 km^2^), “small polygon” (> 25 and ≤ 100 km^2^) and “large polygon” (> 100 km^2^). While month and year defined the start as well as the end of an experiment (two data records could have similar start dates but different end dates), only the study starting year was used to categorise the time of the study. Accurate estimates of the number of collection nights for each individual study (the sampling effort) could not be extracted because only start and end month were recorded and the number of surveys, traps and work-hours were not recorded in the database.

Collection methods were only analysed if they captured species of interest. In the construction of the dataset, mosquito collection methods were categorised as: ‘vector biology sampling’, ‘infection sampling’, ‘human biting rate collection’ and ‘resting collection’ depending on the bionomic metric the data were informing. These categories were not mutually exclusive, so for each data record a collection method could be recorded twice (see also Supplementary File 1). The data frame was manipulated to summarise the combination of collection methods used per data record, ensuring that the location (indoors or outdoors) for each sampling effort was noted. The different collection methods recorded in the database targeting host-seeking or resting mosquitoes were categorised as by Farlow et al.^9^ (Tables 1, 2).

All geo-referenced data records were compiled, summarised by continent, stratified by year and method, and mapped in R Studio. The Mann–Whitney U test analysed within-continent differences between indoor and outdoor sampling methods. To explore trends in the proportional use of host-seeking and resting collections over the entire database time period, a generalised linear model was constructed with ‘proportion host-seeking collections’ as dependent variable and ‘start year of the research’ as independent variable. Running the model with a quasibinomial distribution accounted for overdispersion of the data. The data were summarised by 5-year periods for visualisation. Potential geospatial patterns of collection method use were first studied visually in each of the three regions analysed. Consequently, the two sampling methods used most often that showed at least some degree of clustering were analysed by density-based clustering following the OPTICS algorithm, described in detail by Hahsler et al. (2019)^44^. OPTICS starts with a random data point and provides the order in which new points are explored and added to a cluster. Follow-up ξ-extraction is required to detect clusters of variable density and provide the cluster hierarchy. This means that the OPTICS method can detect clusters within clusters. Afterwards, Moran’s I for spatial autocorrelation tested whether the detected clustering of the analysed sampling methods was significant, or the locations where a sampling method was deployed were spread randomly across a continent/region. Data analyses were performed in R Studio with R statistical software (R version 4.0.2) using the packages ‘tidyverse’, ‘maps’, ‘gganimate’, ‘geosphere’, ‘ape’, ‘psych’, ‘dbscan’ and ‘sjmisc’^45^.

## Supplementary Information

Supplementary Information.

## Data Availability

All data generated or analysed during this study are included in this published article and its supplementary information files. Original databases can also be found in Massey NC, Garrod G, Wiebe A, Henry AJ, Huang Z et al. *Sci Data*. A global bionomic database for the dominant vectors of human malaria. 2016;3:1–13. https://doi.org/10.1038/sdata.2016.14.

## Abbreviations

ABT: Animal-baited trap
Data record: A unique site-collection period-species combination which corresponds with a single row in the original database
DVS: Dominant vector species
HLC: Human landing catch
HBT: Human-baited double net trap
IRS: Indoor residual spraying
LLIN: Long-lasting insecticidal bed net
NMCP: National Malaria Control Program
PSC: Pyrethrum spray collection
WHO: World Health Organisation
